# Decitabine Suspends Human CD34^+^ Cell Differentiation and Proliferation during Lentiviral Transduction

**DOI:** 10.1371/journal.pone.0104022

**Published:** 2014-08-04

**Authors:** Naoya Uchida, Matthew M. Hsieh, Charlotte Platner, Yogen Saunthararajah, John F. Tisdale

**Affiliations:** 1 Molecular and Clinical Hematology Branch, National Heart Lung and Blood Institutes (NHLBI)/National Institute of Diabetes and Digestive and Kidney Diseases (NIDDK), National Institutes of Health (NIH), Bethesda, Maryland, United States of America; 2 Translational Hematology and Oncology Research, Taussig Cancer Institute, Cleveland Clinic, Cleveland, Ohio, United States of America; Southern Illinois University School of Medicine, United States of America

## Abstract

Efficient *ex* *vivo* transduction of hematopoietic stem cells (HSCs) is encumbered by differentiation which reduces engraftment. We hypothesized that inhibiting DNA methyltransferase with decitabine would block differentiation of transduced CD34^+^ cells under cytokine stimulation and thus improve transduction efficiency for engrafting HSCs. Human CD34^+^ cells in cytokine-containing media were treated with or without decitabine for 24 or 48 hours, and then these cells were transduced with a GFP-expressing lentiviral vector. Utilizing decitabine pre-treatment for 48 hours, we observed an equivalent percentage of successfully transduced cells (GFP-positivity) and a higher percentage of cells that retained CD34 positivity, compared to no decitabine exposure. Cell proliferation was inhibited after decitabine exposure. Similar results were observed among CD34^+^ cells from six different donors. Repopulating activity was evaluated by transplantation into NOD/SCID/IL2Rγ^null^ mice and demonstrated an equivalent percentage of GFP-positivity in human cells from decitabine-treated samples and a trend for higher human cell engraftment (measured 20–24 weeks after transplantation), compared to no decitabine exposure. In conclusion, *ex* *vivo* decitabine exposure inhibits both differentiation and proliferation in transduced human CD34^+^ cells and modestly increases the engraftment ability in xenograft mice, while the transduction efficiency is equivalent in decitabine exposure, suggesting improvement of lentiviral transduction for HSCs.

## Introduction

Hematopoietic stem cell (HSC) targeted gene therapy is potentially curative for various hereditary and acquired diseases, and recent clinical trials have demonstrated efficacy in disorders in which a selective advantage is conferred upon corrected cells [1], [2], [3], [4], [5], [6]. However, further improvement of transduction strategies for human HSCs remains necessary before widespread application. To genetically modify HSCs with a therapeutic gene, human CD34^+^ cells are cultured *in* *vitro* with cytokine stimulation and transduced with retrovirus-based vectors, such as γ-retroviral vectors or lentiviral vectors. The inclusion of cytokines is required to maintain repopulating ability of HSCs during *in* *vitro* culture, while overstimulation by higher cytokine concentration or longer *in* *vitro* culture reduces their repopulating ability [7]. Viral vectors achieve their therapeutic effect by integrating into genomic DNA of target cells to stably express a desired gene, but these vectors have a potential risk of mutagenesis by inserting into or near cellular oncogenes [3], [5], [8], [9]. Additionally, the lentiviral vectors have a tendency to be integrated into activated genes (in euchromatin), and transgene expression can be inhibited by DNA methylation in promoter regions [10], [11], [12], [13].

The drug decitabine depletes DNA methyltransferase 1 (DNMT1), which is a key modulator of euchromatin and heterochromatin. This effect has been exploited to induce fetal hemoglobin expression in erythroid cells for patients with sickle cell disease, with the main side effect being leukopenia [14]. In *in* *vitro* culture, decitabine and histone deacetylase (HDAC) inhibitors preserve the stem cell profile of human HSCs and embryonic stem cells (ES cells) [15], [16], [17], [18], [19], [20], [21]. Additionally, these drugs show antileukemia effects in acute myeloid leukemia and myelodysplastic syndrome by relieving aberrant epigenetic gene silencing [22], [23], [24]. These epigenetic modifiers can modulate cell differentiation, proliferation, and transcriptional regulation.

Based on these observations, we hypothesized that decitabine would block differentiation of CD34^+^ cells transduced under cytokine stimulation and while improving transduction efficiency. This hypothesis was tested by evaluating the effects of decitabine on lentiviral transduction and engraftment of human CD34^+^ cells.

## Methods

### Lentiviral vector preparation

The self-inactivating human immunodeficiency virus-1 (HIV-1) based lentiviral vectors were prepared as previously described [25], [26]. We prepared an HIV-1 vector encoding enhanced green fluorescent protein (GFP) under the control of the murine stem cell virus (MSCV) promoter using 4 plasmids; Gag/Pol, Rev/Tat, vesicular stomatitis virus glycoprotein envelope, and HIV-1 vector (pCL20cMpGFP) plasmids [27]. The HIV-1 vector systems were kindly provided by Dr. Arthur Nienhuis (St. Jude Children’s Research Hospital, Memphis, TN, USA) [28], [29]. The viral titers were evaluated by GFP expression in transduced HeLa cells, as previously described [26]. HeLa cells (5×10e4 cells per well) were split into 12-well dishes, and after 24 hours, the cells were transduced with lentiviral vectors in 1 ml of Dulbecco’s modified Eagle media (DMEM) containing 10% fetal bovine serum (FBS) and 8 µg/ml polybrene (Sigma-Aldrich, St. Louis, MO, USA). Three or four days later, GFP expression was detected by flow cytometry (FACSCalibur, BD Biosciences, Franklin Lakes, NJ, USA).

### Lentiviral transduction for human CD34^+^ cells with decitabine exposure

Human CD34^+^ cells were enriched from peripheral blood stem cells mobilized by granulocyte colony-stimulating factor (G-CSF) under a study (03-H-0015) that was approved in 2003 by the Institutional Review Board of the National Heart, Lung, and Blood Institute (NHLBI) and under another study (08-H-0156) that was approved in 2008 by the Institutional Review Board of the National Institute of Diabetes, Digestive, and Kidney diseases (NIDDK) [7], [26]. All patients gave written informed consent for the sample donation and consent documents are maintained in the donor’s medical records. The consent document was approved by the Institutional Review Board prior to study initiation and is reviewed and updated yearly. Human CD34^+^ cells (1×10e5 cells per well) were cultured on fibronectin- (RetroNectin; Takara, Shiga, Japan) coated plates using serum-free X-VIVO10 media (Lonza, Allendale, NJ, USA) containing stem cell factor (SCF), fms-like tyrosine kinase 3 ligand (FLT3L), and thrombopoietin (TPO) (all 100 ng/ml, R&D Systems, Minneapolis, MN, USA) supplemented with decitabine (0.05–0.5 µM, Sigma-Aldrich) in triplicate (N = 3) [12]. After 24 or 48 hours of prestimulation with decitabine, the cells were transduced with a GFP-expressing HIV-1 vector at multiplicity of infection (MOI) 50 without decitabine for 24 hours. After transduction, media were changed to fresh media containing cytokines and decitabine, and 3 days later, both CD34 expression and GFP expression were evaluated by flow cytometry, and both cell counts and viability were evaluated with trypan blue stain. Additional 2 days later, genomic DNA was extracted from the transduced cells, and average vector copy number per cell was evaluated by real time polymerase chain reaction (PCR), as previously described [30]. In addition, we investigated supplement of interleukin-3 (IL3) (20 ngml, R&D Systems) into media during prestimulation and transduction. We used the same donor cells in all experiments except for comparison among CD34^+^ cells from 6 donors.

### Erythroid differentiation from human CD34^+^ cells with lentiviral transduction

Human CD34^+^ cells were prestimulated in X-VIVO10 containing SCF, FLT3L, and TPO supplemented with 0.5 µM decitabine for 48 hours, and the cells were transduced with the GFP-expressing HIV-1 vector without decitabine. After 24-hour transduction, the cells were cultured in erythroid differentiation media containing erythropoietin (2 U/ml) for 1–3 weeks, as previously described [13], [26]. The GFP expression in glycophorin A (GPA)-positive erythroid cells were evaluated by flow cytometry.

### *In vitro* subclonal expansion assay in human CD34^+^ cells with lentiviral transduction

After 48-hour prestimulation with 0.5 µM decitabine, human CD34^+^ cells were transduced with the GFP-expressing HIV-1 vector at MOI 50 for 24 hours (without decitabine). After 2 week culture in X-VIVO10 media containing SCF, FLT3L, and TPO, these cells (100 cells per well) were cultured in 96-well plates in cytokine-free DMEM with 10% FBS for 3–4 weeks to assay the expansion ability of transduced cells [31], [32]. The cell expansion and GFP expression were evaluated by fluorescent microscopy. In addition, we investigated supplement of IL3 into media during prestimulation and transduction.

### Xenograft mouse transplantation of human CD34^+^ cells with lentiviral transduction

We used male NOD/SCID/IL2Rγ^null^ mice (NOD.Cg-*Prkdc^scid^ IL2rg^tm1Wjl^*/SzJ; Jackson Laboratory, Bar Harbor, ME), following the guidelines set out by the Public Health Services Policy on Humane Care and Use of Laboratory Animals under a protocol approved by the Animal Care and Use Committee of the National Heart, Lung, and Blood Institute (NHLBI) [7], [13]. We transduced human CD34^+^ cells (2×10e6 cells per mouse) with the GFP-expressing HIV-1 vector at MOI 50 after 48-hour prestimulation with 0.5 µM decitabine, and these cells were injected into NOD/SCID/IL2Rγ^null^ mice 2 days after sublethal dose of busulfan injection (35 mg/kg, Busulfex; PDL BioPharma, Redwood City, CA, USA). Human CD45 expression (human CD45-PE antibody, clone HI30; BD Biosciences) and GFP expression were evaluated in peripheral blood cells for 6 months after transplantation. In addition, we investigated supplement of IL3 into media during prestimulation and transduction.

### Statistical analysis

Statistical analyses were performed using the JMP 9 software (SAS Institute Inc., Cary, NC, USA). The averages in various conditions were evaluated by Dunnett’s test (one-way analysis of variance for one control). Two averages were evaluated by the student’s *t*-test. Standard errors of the mean are shown as error bars in all figures except for comparison among 6 donors’ cells, in which the standard deviations were used and evaluated by *F*-test. A p value of <0.01 or 0.05 was deemed significant.

## Results

### Decitabine exposure preserved CD34 expression in cultured human CD34^+^ cells during lentiviral transduction

**Figure 1:**
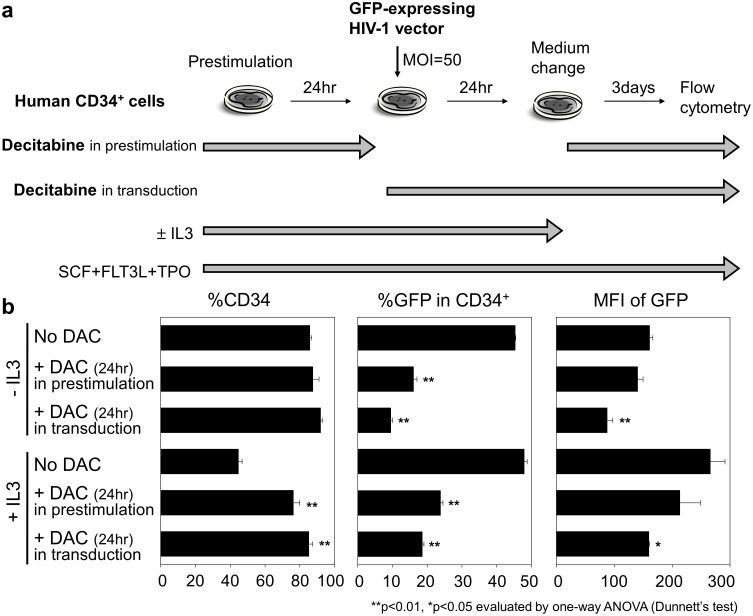
Decitabine reduced GFP-positive rates (%GFP) in transduced human CD34^+^ cells. (a). Human CD34^+^ cells were cultured in serum-free media containing cytokines (SCF, FLT3L, and TPO). After 24 hours of prestimulation, the cells were transduced with GFP-expressing HIV-1 based lentiviral vectors for 24 hours. Decitabine (0.5 µM) was added into media during either prestimulation (24 hours) or transduction (24 hours). After transduction, media were changed to fresh media containing cytokines and decitabine, and 3 days later, both CD34 expression and GFP expression were evaluated by flow cytometry. In addition, we investigated supplement of IL3 into media during prestimulation and transduction. (b). The %GFP in CD34^+^ cells was decreased by decitabine exposure in both prestimulation and transduction. Decitabine treatment led to higher CD34-positive rates (%CD34) when IL3 was added. We tested statistical significance of data in both decitabine exposure groups, compared to no decitabine exposure. DAC: decitabine, SCF: stem cell factor, FLT3L: fms-like tyrosine kinase 3 ligand, TPO: thrombopoietin, IL3: interleukin-3, MFI: mean fluorescence intensity, and MOI: multiplicity of infection.

To evaluate decitabine effects on CD34^+^ cell transduction, human CD34^+^ cells were cultured in serum-free media containing SCF, FLT3L, and TPO (Figure 1a). After 24 hours of prestimulation, CD34^+^ cells were transduced with an HIV-1 based lentiviral vector encoding GFP under the control of the MSCV promoter for 24 hours. (The same vector constructs were used in all experiments in this research.) Decitabine (0.5 µM) was added into media during either prestimulation (24 hours) or transduction (24 hours). After transduction, media were changed to fresh media containing cytokines and decitabine, and 3 days later, both CD34 expression and GFP expression were evaluated by flow cytometry. In addition, we investigated supplementation with IL3 during prestimulation and transduction.

IL3 supplementation decreased CD34-positive rates (%CD34), consistent with promoting differentiation; this trend was reversed with 24-hour decitabine exposure during both prestimulation and transduction (p<0.01, Figure 1b left panel). The GFP-positive rates (%GFP) in CD34^+^ cells were also decreased by decitabine exposure during both prestimulation and transduction (p<0.01, Figure 1b, center panel). These data showed that decitabine suspended cell differentiation induced by IL3, and adding decitabine during prestimulation or transduction was equivalent.

**Figure 2:**
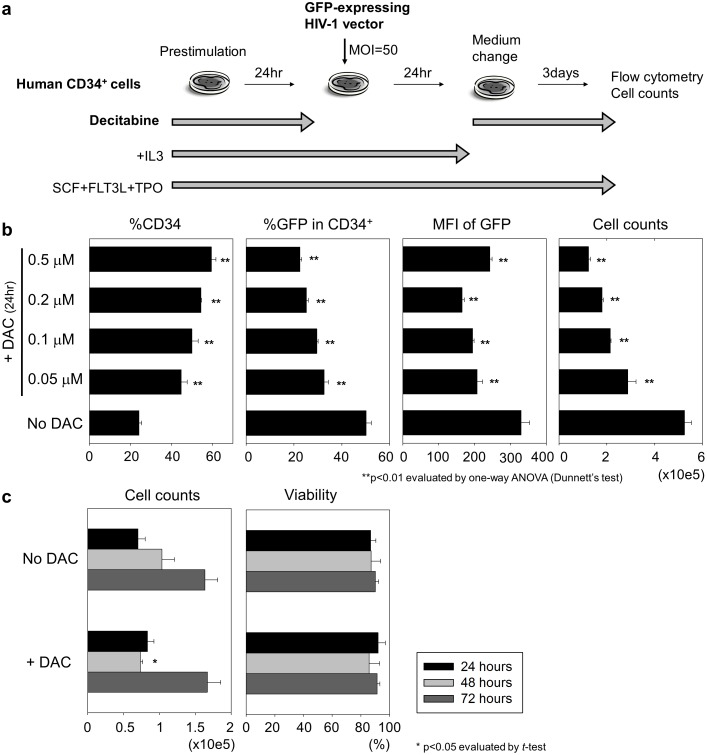
Decitabine increased CD34 expression and reduced cell proliferation in transduced human CD34^+^ cells. (a). To further investigate the decitabine effects on transduction efficiency for human CD34^+^ cells, we added increasing dose of decitabine (0.05, 0.1, 0.2, and 0.5 µM) into media during 24-hour prestimulation (SCF, FLT3L, TPO, and IL3). The GFP expression, CD34 expression, and cell counts were evaluated 4 days after transduction. (b). We observed dose-dependent effects of decitabine exposure, which resulted in higher %CD34, lower %GFP, and lower cell counts. (c). We evaluated cell counts and viability of human CD34^+^ cells at 24, 48, and 72 hours after decitabine exposure (0.5 µM) with cytokine stimulation (SCF, FLT3L, TPO, and IL3) without transduction. Decitabine exposure resulted in lower cell counts at 48 hours after exposure; however, equivalent cell counts were observed at 24 hours and 72 hours after exposure. Decitabine exposure did not decrease viability at any time points. We tested statistical significance of data in all decitabine exposure groups, compared to no decitabine exposure.

To further investigate the decitabine effects on transduction, we added increasing concentrations of decitabine (0.05, 0.1, 0.2, and 0.5 µM) into media during 24-hour prestimulation with SCF, FLT3L, TPO, and IL3. At 4 days after transduction, we evaluated cell counts as well as CD34 expression and GFP expression (Figure 2a). We observed dose-dependent effects of decitabine exposure, which resulted in higher %CD34 (p<0.01, Figure 2b first panel), lower %GFP (p<0.01, Figure 2b second panel), and lower cell counts (p<0.01, Figure 2b fourth panel).

To investigate decitabine effects on proliferation of human CD34^+^ cells, we evaluated both cell counts and viability of CD34^+^ cells at 24, 48, and 72 hours after decitabine exposure (0.5 µM) with cytokine stimulation (SCF, FLT3L, TPO, and IL3) without transduction. 48-hour decitabine exposure resulted in lower cell counts (p<0.05, Figure 2c left panel), compared to no decitabine exposure, while equivalent cell counts were observed at 24 and 72 hours after decitabine exposure. Decitabine exposure did not decrease viability at any time points (Figure 2c right panel). These data suggest that decitabine inhibits proliferation of human CD34^+^ cells from 24 hours to 48 hours, and the proliferation starts from 48 hours to 72 hours following decitabine exposure.

**Figure 3:**
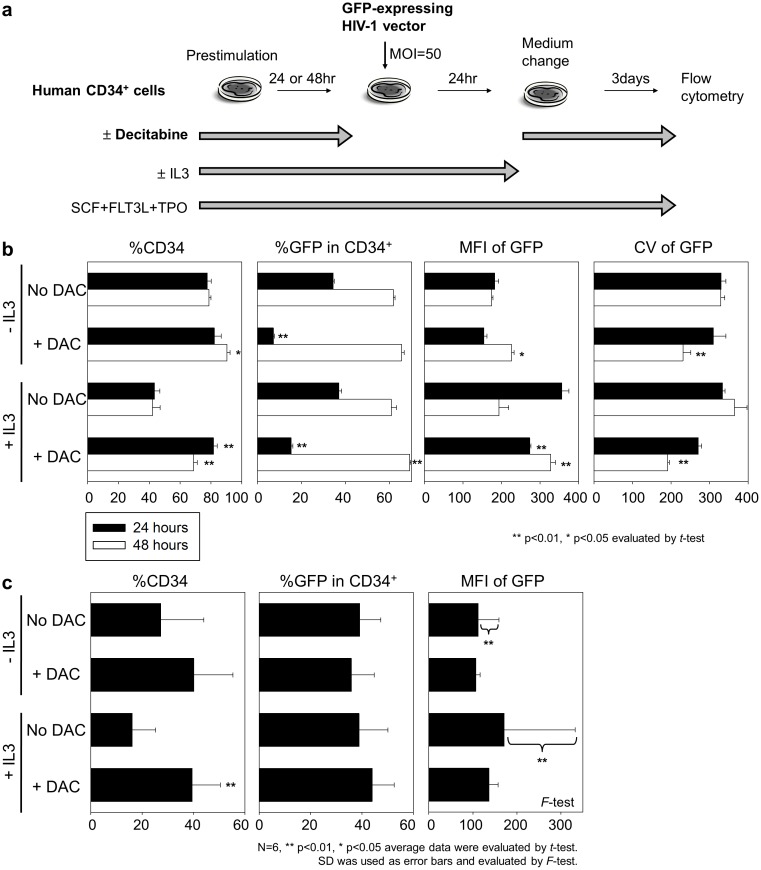
48-hour decitabine exposure resulted in equivalent %GFP in transduced human CD34^+^ cells. (a). To evaluate whether transduction efficiency is changed by interval of time between decitabine exposure and transduction, we compared %GFP between 24-hour and 48-hour prestimulation. Human CD34^+^ cells were cultured in serum-free media containing cytokines (SCF, FLT3L, and TPO) supplemented with decitabine (0.5 µM). At 24 or 48 hours after starting exposure with or without decitabine, the cells were transduced with GFP-expressing HIV-1 based lentiviral vectors without decitabine for 24 hours. In addition, we investigated supplement of IL3 into media during prestimulation and transduction. (b). After 48-hour prestimulation, decitabine exposure resulted in higher %CD34, equivalent %GFP, and lower coefficient of variation (CV) of GFP intensity, while %GFP decreased in 24-hour decitabine exposure. (c). In transduction of CD34^+^ cells obtained from six healthy donors, average data revealed similar trends of higher %CD34 and equivalent %GFP after 48-hour decitabine exposure (0.5 µM). In addition, the decitabine exposure reduced standard deviations (SD) of GFP intensity among transduced CD34^+^ cells from six individuals (right panel). We tested statistical significance of data in all decitabine exposure groups, compared to no decitabine exposure.

We hypothesized that transduction efficiency might be improved by longer decitabine exposure. Therefore, we transduced human CD34^+^ cells 24 or 48 hours after exposure with or without 0.5 µM decitabine (Figure 3a). At 24 hours, %GFP was reduced by decitabine exposure, with or without IL3 supplementation (p<0.01, Figure 3b second panel). However, after 48 hours, decitabine exposure resulted in equivalent %GFP and higher %CD34 (p<0.05), compared to no decitabine exposure (Figure 3b first and second panels). In addition, lower coefficient of variation (CV) of GFP intensity was observed after 48-hour decitabine exposure (p<0.01, Figure 3b fourth panel).

These data demonstrated that decitabine suspends differentiation and proliferation during transduction of human CD34^+^ cells. Decitabine exposure for 48 hours results in equivalent transduction efficiency, while 24-hour exposure reduces the transduction efficiency.

### Decitabine exposure reduced the variability of GFP intensity among CD34^+^ cells from six different donors

We evaluated decitabine effects on lentiviral transduction using CD34^+^ cells obtained from six healthy donors (Figure 3c). Average data revealed similar trends of higher %CD34 and equivalent %GFP after 48-hour decitabine exposure (0.5 µM). In addition, decitabine reduced the standard deviations of GFP intensity (p<0.01) among the CD34^+^ cells from six individuals (Figure 3c right panel). These data indicated that decitabine could reduce the variation of transgene expression levels in transduced human CD34^+^ cells.

### Post-transduction decitabine exposure reduced the variability of GFP intensity in transduced CD34^+^ cells

**Figure 4:**
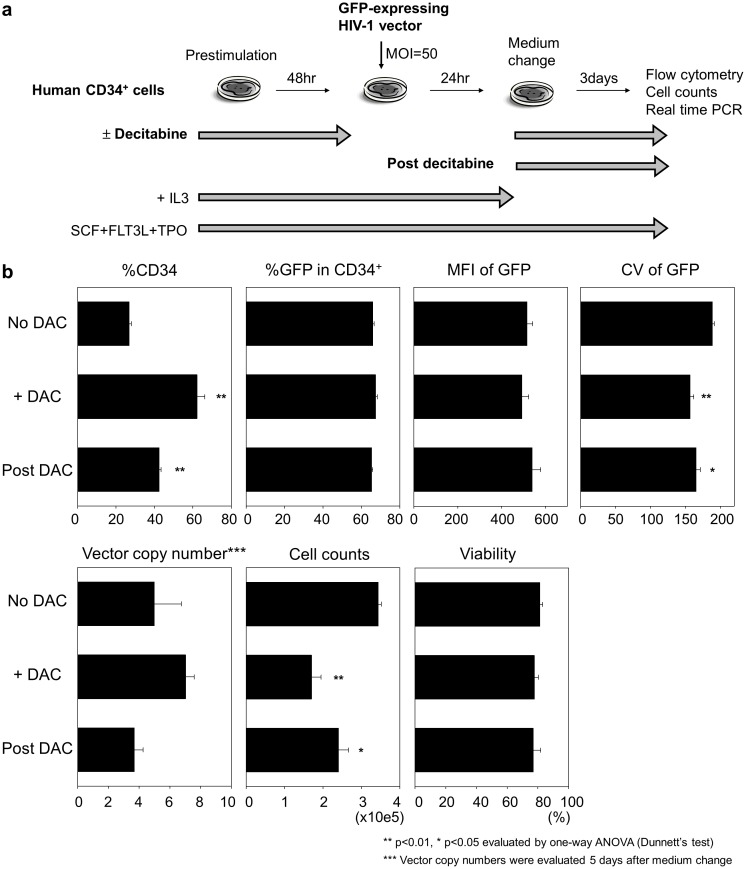
Post-transduction decitabine exposure reduced variability of GFP intensity in transduced CD34^+^ cells. (a). To evaluate whether decitabine exposure affects internal promoter activity in lentiviral vectors to drive GFP expression, human CD34^+^ cells were exposed to decitabine (0.5 µM) following lentiviral transduction (Post DAC) with 48-hour prestimulation (SCF, FLT3L, TPO, and IL3), and we analyzed GFP expression by flow cytometry and average vector copy number per cell by real time polymerase chain reaction (PCR). (b). Post-transduction decitabine exposure resulted in equivalent %GFP, GFP intensity, and vector copy number, while lower CV of GFP intensity was observed in post-transduction decitabine exposure, compared to no decitabine exposure. The similar decitabine effects were detected when CD34^+^ cells were exposed to decitabine before and after transduction. In addition, higher %CD34, lower cell counts, and equivalent viability were observed in both decitabine exposure groups. We tested statistical significance of data in all decitabine exposure groups, compared to no decitabine exposure.

To evaluate whether decitabine exposure affects not only lentiviral transduction efficiency but also the internal promoter activity to drive GFP expression, human CD34^+^ cells were exposed to decitabine (0.5 µM) following lentiviral transduction with 48-hour prestimulation (SCF, FLT3L, TPO, and IL3), and we analyzed GFP expression by flow cytometry and average vector copy number per cell by real time PCR (Figure 4a). Post-transduction decitabine exposure resulted in equivalent %GFP, GFP intensity, and vector copy number (Figure 4b second, third, and fifth panels), while lower CV of GFP intensity was observed in post-transduction decitabine exposure (p<0.05), compared to no decitabine exposure (Figure 4b fourth panel). The similar decitabine effects were detected when CD34^+^ cells were exposed to decitabine before and after transduction (Figure 2b second, third, fourth, and fifth panels). In addition, higher %CD34 (p<0.01, Figure 4b first panel), lower cell counts (p<0.05 and 0.01, respectively, Figure 4b sixth panel), and equivalent viability (Figure 4b seventh panel) were observed in both decitabine exposure groups, compared to no decitabine exposure. These data suggest that lower variability of GFP intensity is mediated by modification of the promoter activity but not by vector integration, while %GFP was mainly affected by lentiviral transduction efficiency.

### Decitabine exposure decreased subclonal expansion in transduced CD34^+^ cells

**Figure 5:**
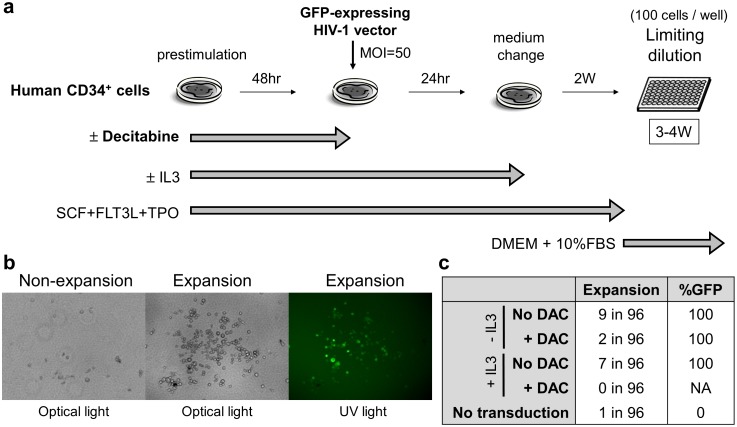
Decitabine reduced subclonal expansion of transduced CD34^+^ cells. (a). We performed a limiting dilution of human CD34^+^ cells which were transduced with lentiviral vectors after 48-hour decitabine exposure (0.5 µM), and then cultured in 96-well plates for 3–4 weeks to assay the expansion ability of transduced cells. (b). We detected subclonal expansion of GFP-positive cells that had originated from the transduced CD34^+^ cells. (c). The expansion rates were reduced by decitabine exposure under cytokine prestimulation without IL3 (2.1% vs. 9.4%) and with IL3 (0.0% vs. 7.3%). FBS: fetal bovine serum, NA: not applicable.

We next performed a limiting dilution experiment of human CD34^+^ cells, in which CD34^+^ cells were transduced with lentiviral vectors after 48-hour decitabine exposure (0.5 µM), and then bulk CD34^+^ cells were cultured in 96-well plates (100 cells per well) for 3–4 weeks to assay the expansion potential in cytokine-free DMEM media with 10% FBS (Figure 5a). Even though we did not sort GFP-positive cells, we detected subclonal expansion of GFP-positive cells but not GFP-negative cells, which had originated from the transduced CD34^+^ cells (Figure 5b). Decitabine reduced the expansion rates under both prestimulation conditions of standard cytokines (2.1% vs. 9.4%) and IL3 supplement (0.0% vs. 7.3%) (Figure 5c). These results suggest that lentiviral transduction could induce subclonal expansion of differentiated cells which were derived from transduced CD34^+^ cells, and the subclonal expansion of transduced cells could be reduced by 48-hour decitabine exposure for CD34^+^ cells before transduction.

### Decitabine exposure promotes erythroid differentiation in transduced CD34^+^ cells

**Figure 6:**
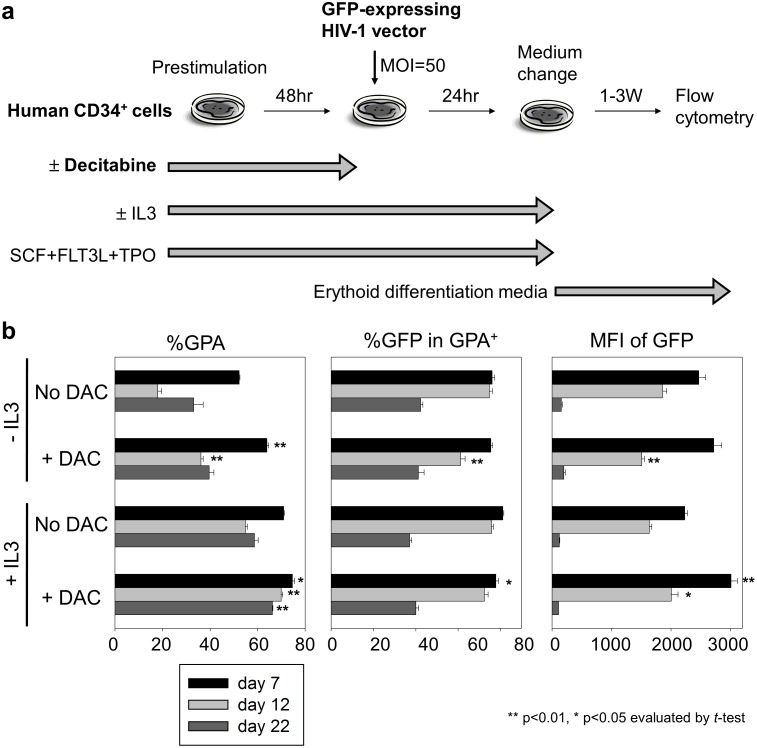
Decitabine promotes efficient erythroid differentiation from transduced CD34^+^ cells. (a) To evaluate decitabine effects on erythroid differentiation and transduction efficiency, we transduced human CD34^+^ cells with a GFP-expressing HIV-1 vector following 48-hour prestimulation with 0.5 µM decitabine. After 24-hour transduction (without decitabine), the cells were differentiated into erythroid cells. (b) Both %GFP and GFP intensity in GPA-positive cells gradually decreased during the erythroid differentiation. The glycophorin A-positive rates (%GPA) were increased by decitabine exposure, while equivalent %GFP was observed. We analyzed data in decitabine exposure groups, compared to no decitabine exposure.

To evaluate decitabine effects on erythroid differentiation from transduced CD34^+^ cells, we transduced human CD34^+^ cells with a GFP-expressing HIV-1 vector after a 48-hour exposure with 0.5 µM decitabine (Figure 6a). Following 1–3 weeks of erythroid differentiation using erythropoietin, we evaluated GPA-positive rates and %GFP by flow cytometry. Both %GFP and GFP intensity in GPA-positive cells gradually decreased during the procedure of erythroid differentiation (Figure 6b). Decitabine exposure increased GPA-positive rates (p<0.05 at all time points except day 22 without IL3, Figure 6b left panel), while equivalent %GFP in GPA-positive cells was observed (Figure 6b, center panel). These results suggest that decitabine exposure during a 48-hour prestimulation can promote erythroid differentiation with erythropoietin exposure for 1–3 weeks.

### Decitabine exposure *ex* *vivo* modestly increased human cell engraftment in xenograft mice

**Figure 7:**
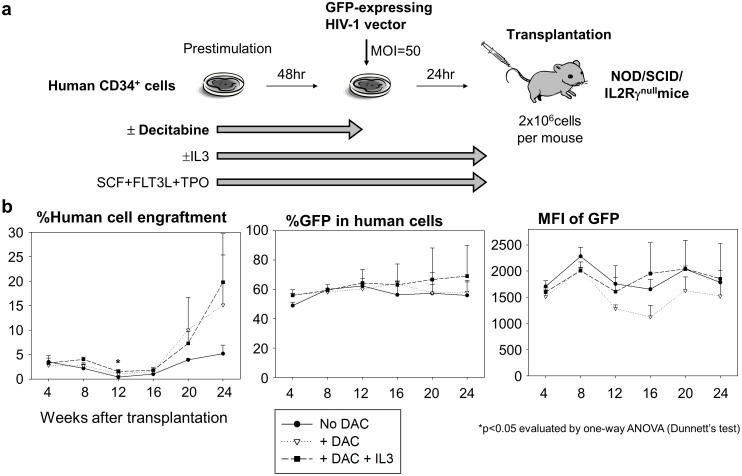
Decitabine modestly increased human cell engraftment in humanized xenograft mice. (a). To evaluate decitabine effects on long-term progenitor cells *in* *vivo*, we transduced human CD34^+^ cells after 48-hour prestimulation with and without decitabine (0.5 µM), and injected into sublethally-conditioned immunodeficient mice. (b). In 48-hour *ex* *vivo* decitabine exposure either with or without IL3, we observed equivalent %GFP and a tendency towards higher human cell engraftment at 20–24 weeks after transplantation, compared to no decitabine exposure (48-hour prestimulation without IL3).

To evaluate decitabine effects on long-term hematopoietic repopulating cells *in* *vivo*, we transduced human CD34^+^ cells after 48-hour prestimulation with decitabine, and injected into sublethally-conditioned immunodeficient mice (Figure 7a). Human CD45 expression and %GFP in peripheral blood cells were evaluated for 6 months after transplantation. 48-hour *ex* *vivo* decitabine exposure either with or without IL3 showed equivalent %GFP in human cells and a tendency towards higher human cell engraftment at 20–24 weeks after transplantation, compared to no decitabine exposure with 48-hour prestimulation (without IL3) (Figure 7b). These data suggest that 48-hour decitabine exposure *ex* *vivo* resulted in equivalent transduction efficiency for hematopoietic repopulating cells, while this exposure might increase the engraftment ability.

## Discussion

In HSC gene therapy, human CD34^+^ cell culture with cytokine stimulation *ex* *vivo* is required for efficient transduction with lentiviral vectors; however, overstimulation with cytokines reduces human cell engraftment in xenograft mice [7]. Decitabine and other “chromatin-relaxing” drugs (such as histone deacetylase inhibitors) were reported to maintain stem cell capacity of HSCs and ES cells in *in* *vitro* culture [15], [16], [17], [18], [19], [20], [21], [33], [34], [35]. In this study, we confirmed that decitabine has several beneficial effects in the transduction process. First, decitabine maintains CD34 expression of human CD34^+^ cells during the entire lentiviral transduction process (Figure 3), whether supplemented during prestimulation or transduction (Figure 1). Decitabine also partially prevents IL3-induced cell differentiation by maintaining CD34 expression (Figures 1, 2, 3, and 4). Additionally, 48-hour decitabine exposure during 3 day *ex* *vivo* culture revealed a tendency to increase engraftment ability of human CD34^+^ cells in xenograft mice 6 months after transplantation (Figure 7).

There are additional effects exerted by decitabine that were not expected during the lentiviral transduction process. We observed that decitabine exposure reduced the variability of GFP intensity in bulk CD34^+^ cells, evidenced by the lower CV of the GFP mean fluorescent intensity (MFI) in a single donor of CD34^+^ cells and the smaller standard deviations of the GFP MFI from 6 donors (Figures 3 and 4). The lower CV of GFP intensity was observed even in post-transduction decitabine exposure (Figure 4), suggesting that this effect was mainly mediated by modification of the MSCV promoter activity to drive GFP expression in the vector construct.

Since some of the variation from transduction might result from different stages of cell differentiation, when decitabine “restricts” or “halts” cell differentiation, variation in differentiation and subsequent transduction would be reduced. This finding is consistent with reduced expansion during the expanded culture of 3–4 weeks after transduction (Figure 5). Additionally, within each well in the 96-well plate containing 100 human CD34^+^ cells, there was a dramatic reduction in number of wells in which the cells expanded after cytokine-free culture. Interestingly, the subclonal expansion was observed in only GFP-positive cells (except one subclone from non-transduced CD34^+^ cells), suggesting that lentiviral integration could induce cytokine-independent expansion ability which might be reduced by decitabine exposure during prestimulation.

Adding IL3 into prestimulation and transduction media (including SCF, FLT3L, and TPO) significantly reduced %CD34 in human CD34^+^ cell culture, this trend was reversed with either 24-hour decitabine exposure (Figures 1, 2, and 3) or 48-hour decitabine exposure (Figures 3 and 4). However, we previously demonstrated that IL3 supplementation into CD34^+^ cell culture media decreases CD34 expression during *in* *vitro* culture with lentiviral transduction, but it did not change engraftment ability of CD34^+^ cells in xenograft mice [7]. This finding can be explained by IL3 inducing more expansion of differentiated cells from CD34^+^ cells but not reducing repopulation potential of hematopoietic repopulating cells. These results suggest that IL3 is not necessary for most lentiviral transduction in hematopoietic repopulating cells, with or without decitabine exposure.

Decitabine exposure during a 48-hour prestimulation revealed equivalent %GFP in lentivirally transduced CD34^+^ cells *in* *vitro* and in xenograft mice, compared to no decitabine exposure control. However, with a 24-hour prestimulation, %GFP *in* *vitro* was decreased by decitabine exposure (Figures 3 and 7). We previously demonstrated that transduction efficiency in human CD34^+^ cells was increased by cell proliferation [36], [37]. Decitabine exposure reduces expansion of CD34^+^ cells from 24 hours to 48 hours post exposure and induces the expansion from 48 hours to 72 hours post exposure (Figure 2c), and this effect could contribute to transduction efficiency for CD34^+^ cells with decitabine exposure.

Decitabine can induce fetal hemoglobin production in red blood cells in patients with sickle cell disease [14], [38], [39], [40]. In these studies, decitabine reversed the suppressors of fetal hemoglobin in erythroid cells. In current study, we demonstrated that 48-hour decitabine exposure of human CD34^+^ cells results in higher GPA-positive rates 1–3 weeks after erythroid differentiation (Figure 6). This property of not only maintaining stem cell capacity but also erythroid differentiation potential from human CD34^+^ cells is desirable for gene therapy application in red cell disorders.

In summary, decitabine maintained CD34 expression during lentiviral transduction, reduced transduction variability of human CD34^+^ cells, and modestly increased the engraftment potential of transduced hematopoietic repopulating cells in xenograft mice. These observations are helpful in developing novel strategies to optimize lentiviral transduction of human CD34^+^ cells.
